# Effect of Commuter Time on Emergency Medicine Residents

**DOI:** 10.7759/cureus.2056

**Published:** 2018-01-12

**Authors:** Christopher Sampson, Marc Borenstein

**Affiliations:** 1 Emergency Medicine, University of Missouri Columbia; 2 Emergency Medicine, Brookdale University Hospital and Medical Center

**Keywords:** emergency medicine residents, wellness, driving

## Abstract

Background

The impact of resident work hours on resident well-being and patient safety has long been a controversial issue.

Objectives

What has not been considered in resident work hour limitations is whether resident commuting time has any impact on a resident's total work hours or well-being.

Methods

A self-administered electronic survey was distributed to emergency medicine residents in 2016.

Results

The survey response was 8% (569/6828). Commuter time was 30 minutes or less in 70%. Two residents reported a commuter time of 76 to 90 minutes and one resident had a commuter time of 91 to 105 minutes. None reported commuter times greater than 105 minutes. Of most concern was that 29.3% of the residents reported falling asleep while driving their car home from work. We found 12% of respondents reporting being involved in a car collision while commuting. For residents with commute times greater than one hour, 66% reported they had fallen asleep while driving. When asked their opinion on the effect of commute time, those with commute times greater than one hour (75% of residents) responded that it was detrimental.

Conclusions

While the majority of emergency medicine residents in this survey have commuter times of 30 minutes or less, there is a small population of residents with commuter times of 76 to 105 minutes. At times, residents whose commute is up to 105 minutes each way could be traveling a total of more than 3.5 hours for each round trip. Given that these residents often work 12-hour shifts, these extended commuter times may be having detrimental effects on their health and well-being.

## Introduction

The impact of work hours on resident well-being and patient safety have long been a controversial issue. The death of Libby Zion in 1984 following her admission to the hospital less than 20 hours after her evaluation from the emergency department (ED) led to an in-depth look at resident physician work hours [1].

In 2003, the Accreditation Council for Graduate Medical Education (ACGME) instituted the common duty hour standards, which included an 80-hour workweek averaged over four weeks and an adequate rest period, which was defined as 10 hours of rest per duty period. For emergency medicine, this was further defined such that residents may work no more than 12 continuous hours with an equivalent amount of time off between scheduled work periods. An emergency medicine resident may work no more than 60 scheduled hours in an ED and no more than 72 duty hours in total per week [2]. What has not been considered is whether resident commuting time has any impact on a resident’s workweek, well-being, and/or rest time and if resident commuting time should be considered in calculating total resident work hours. Barger et al. found that extended duration work shifts, defined as greater than 24 hours, posed safety hazards for interns. The risk of falling asleep while driving or while stopped in traffic was also increased when working five or more extended shifts [3].

According to the United States Census Bureau 2009 Report, workers took an average of 25.1 minutes to get to work [4].

There has been research looking into the effect of night-shift work and car crashes. Huffmyer et al. recently found that anesthesia residents who worked six consecutive night shifts had greater difficulty controlling speed and driving performance in a driving simulator session lasting 55 minutes [5]. Lee et al. reported that night-shift work increases driving drowsiness and degrades driving performance [6]. These factors increase the risk of near-crash driving events. In their study, they found that subjective reports and objective indices of drowsiness became prominent within the first 15 to 30 minutes of driving. They also found that driving for more than 45 minutes after a night shift was likely to increase drowsiness-related impairment [6].

Steele et al. reported a higher number of motor vehicle collisions and near-crashes among emergency medicine residents driving home after a night shift [7]. Approximately 74% of the collisions and 80% of the near-crashes occurred following a night shift. This was compared to day shifts where much lower incidences of motor vehicle collisions and near-crashes were found (12% vs. 7%). Shift time was not considered in this study [7].

We sought to investigate resident commuter time, methods of commuting, and the potential consequences of the extended commute.

## Materials and methods

A self-administered electronic survey was generated using SurveyMonkey (www.surveymonkey.com) and distributed to 174 allopathic emergency medicine program directors on a national academic listserv in 2016. Program directors were asked to distribute the survey link among their residents. Responses were collected for four weeks. Participation was voluntary. The study received institutional board review approval (#1210314).

All emergency medicine residents were eligible to participate. The survey was released in May 2016 to make post graduate year (PGY) one residents eligible since they would have completed 10 months of residency. The survey instrument consisted of 12 multiple-choice questions (Table 1).

**Table 1:** Survey questions sent to emergency medicine residents

Survey Questions
What Region is Your Residency Located in?
Year You Are In Residency?
What setting is your hospital?
“Commuter time” is defined as travel to work one way and time must be estimated as daily average time taking into account occasional prolonged time due to bad weather, rush hours, construction delays, etc. What is your commuter time from leaving home to arrival at the hospital?
Number of times per week that commuting trip is taken on average (round trip would be two times).
Does your commuting time change based on rotation locations?
Main method of commuting?
Have you ever fallen asleep while driving your car home?
Have you ever used your GME (Graduate Medical Education) assistance for ride home (taxi, scooter guy)?
What are your shift times?
To what degree do you perceive your commute to have an adverse effect on your wellness/level of fatigue?
Have you ever been in a car collision driving to or from work?

Associations of shift time by commute time and commute method by commute time were studied. Associations of commute time by the incidence of falling asleep while driving and commute time by car collision incidence was calculated using a chi-square analysis using SAS version 9.4 (SAS Institute Inc., Cary NC). Association of commuter time with effect on well-being was also calculated by a chi-square analysis.

## Results

The survey response rate was 8% (569/6828) based on ACGME data of the number of emergency medicine residents in the United States [8]. Response rates were fairly equal over PGY1 to PGY3 (Table 2).

**Table 2:** Demographic information of respondents to survey by residency region, post graduate year, hospital setting, and shift length

Residency Region			n	%
	Northeast		185	32.51%
	Mid-Atlantic		44	7.73%
	Midwest		157	27.59%
	Mountain		1	0.18%
	South		109	19.16%
	West		73	12.83%
Residency Year				
	PGY1		190	33.39%
	PGY2		170	29.88%
	PGY3		135	23.73%
	PGY4		74	13.00%
Hospital Setting				
	Urban		469	82.43%
	Suburban		87	15.29%
	Rural		13	2.28%
Shift Times				
	8 hours		85	14.99%
	10 hours		148	26.10%
	12 hours		165	29.10%
	Other		169	29.81%

Commuter time was found to be 30 minutes or less in 70% of respondents. Sixteen percent of residents reported a commuter time of 31-45 minutes and 11% reported 46-60 minutes. Two residents (0.4%) reported a commuter time of 76-90 minutes and one resident (0.2%) had a commuter time of 91-105 minutes. None reported commuter times greater than 105 minutes. The train was the method of commuting for the resident who reported commute times of 91-105 minutes; this resident also reported working 12-hour shifts. Of the two residents with 76-90 minutes commute times, one commuted by car and the other by train. The former worked eight- and 12-hour shifts and responded yes to falling asleep. The latter worked various shift times (Table 3, Table 4).

**Table 3:** Main method of commuting by respondents

Main Method of Commuting	n	%
Car	415	73%
Bicycle	62	11%
Walk	38	7%
Train	41	7%
Bus	8	1%
Private Driver	5	1%

**Table 4:** Commuter time

Commuter Time	n	%
0-15 min	196	34%
16-30 min	204	36%
31-45 min	91	16%
46-60 min	62	11%
61-75 min	13	2%
76-90 min	2	0.40%
91-105 min	1	0.20%
106-120 min	0	0%
> 120 min	0	0%

Commuter time was defined as the time for one trip from work to home. Most concerning was the 29.3% (n=166) that reported falling asleep while driving their car home from work. Car collisions while commuting were reported in 12%, a higher incidence when compared with Steele et al., who reported only 8% [7].

When looking at the association between shift times and commuting times (Table 5), we found that most residents worked at least 10-hour shifts (n=313, 71.95%). Most residents had 30 minutes or shorter commute times (n=311, 71.49%). Almost half of the residents surveyed worked at least 10-hour shifts and had a 30-minute or less commute (n=205, 47.13%).

**Table 5:** Association of shift time and commuter time

	0-15 min	16-30 min	31-45 min	46-60 min	> 61 min	Total
8 hours	38	33	9	5	0	85
9 hours	18	17	1	1	0	37
10 hours	54	59	21	12	2	148
12 hours	53	39	34	30	9	165
Total	163	148	65	48	11	435

When investigating the association between commute method and commute time, it was found that overall 73% residents commute by car and 97% of residents have commute times within one hour regardless of the method used.

When looking at the association of commute time and incidence of falling asleep while driving, residents who drove for a longer time were more likely to fall asleep. When commute times were greater than one hour, 66% (6/9) reported they had fallen asleep while driving. When asked about car collisions (Table 6), those who drove for a longer time were more likely to have a car collision. Of those who commuted for more than one hour by car, 44% (4/9) reported they had been in a car collision.

**Table 6:** Association of resident’s commuter time and motor vehicle collision *P *< .0001 for the chi-square test of association

	No	Yes
0-15 min	138	5
16-30 min	134	19
31-45 min	53	11
46-60 min	26	18
> 61 min	5	4

When asked their opinion on the effect of commute time on personal well-being, those with commute times greater than one hour (75%; 9/12) responded it was detrimental (Table 7).

**Table 7:** Association of commute time with self-reported feeling of effect on well-being *P *< .0001 for the chi-square test of association

	Negative	Neutral	Positive
0-15 min	5	85	93
16-30 min	8	99	50
31-45 min	6	23	6
46-60 min	24	5	11
> 61 min	9	0	3

## Discussion

As seen in the data presented here, most residents have a short commute time of 30 minutes or less. This is similar to the national commuting data of 25 minutes. What is more concerning is that the 30% of residents who answered this survey had an extended commute time of greater than 30 minutes and possibly up to 105 minutes. If one was working a 12-hour shift and had a 105-minute commute each way, the resident would be commuting for a total of 210 minutes per shift. Adding this on to a 12-hour shift means that the resident would have only 8.5 hours off between shifts for rest and recovery time. If one was to factor in basic daily actions, such as eating and grooming/showering, a resident working a 12-hour shift with a commute time greater than one hour each way would have reduced and possibly insufficient sleep time especially when the recommended sleep is seven to nine hours per day [9]. Given that almost 30% of respondents reported falling asleep while driving, a potential reduction in rest/sleep and recovery time is very concerning for residents who may be commuting more than two hours/day, especially if the resident is driving. Not surprisingly, increased commuter times were associated with a statistically significant decrease in well-being.

This study highlights an area that is often not considered but could potentially be playing an important role in resident wellness. Extended commute times can affect total sleep time, non-work-related time, and the time one needs to decompress. Given the importance of commuting time and its potential implications, one questions whether residency accrediting bodies should consider this data.

Limitations in this study were similar to those in the Steele et al. study of emergency medicine residents and car collisions. These limitations are primarily selection bias and low survey response rate. Reasons for a low response rate are not clear but may have included surveys not having been forwarded to residents by their leadership team. Recall bias regarding falling asleep or having experienced a near-crash driving event is another limitation.

## Conclusions

While the majority of emergency medicine residents surveyed have commuter times of 30 minutes or less, there is a small population of residents with commuter times of 76 to 105 minutes. Given that these residents often work 12-hours shifts, those whose commute is up to 105 minutes each way could be traveling a total round trip of more than 3.5 hours to/from work. These extended commuter times may be having detrimental effects on resident health and well-being. We believe that further research is warranted to investigate these areas and the impact of commuting on resident work hour times. Future efforts may also focus on residence location and commuting time to improve resident wellness.
